# Hydrogen Sulfide and Silicon Together Alleviate Chromium (VI) Toxicity by Modulating Morpho-Physiological and Key Antioxidant Defense Systems in Chickpea (*Cicer arietinum* L.) Varieties

**DOI:** 10.3389/fpls.2022.963394

**Published:** 2022-07-22

**Authors:** Deepti Singh, Chandan Kumar Singh, Manzer H. Siddiqui, Saud Alamri, Susheel Kumar Sarkar, Abhishek Rathore, Saroj Kumar Prasad, Dharmendra Singh, Nathi Lal Sharma, Hazem M. Kalaji, Adam Brysiewicz

**Affiliations:** ^1^Department of Botany, Meerut College, Meerut, India; ^2^Division of Genetics, ICAR-Indian Agricultural Research Institute, New Delhi, India; ^3^Department of Botany and Microbiology, College of Science, King Saud University, Riyadh, Saudi Arabia; ^4^Division of Design of Experiments (DE), ICAR-Indian Agricultural Statistics Research Institute, ICAR Library Avenue, Pusa, New Delhi, India; ^5^Regional Breeding Informatics Lead, Excellence in Breeding Platform (EiB)-CIMMYT Building ICRISAT Campus, Patancheru, Hyderabad, India; ^6^Department of Agronomy, Institute of Agricultural Sciences, Banaras Hindu University, Varanasi, India; ^7^Department of Plant Physiology, Institute of Biology, Warsaw University of Life Sciences SGGW, Warsaw, Poland; ^8^Institute of Technology and Life Sciences-National Research Institute, Falenty, Poland

**Keywords:** chromium, hydrogen sulfide, silicon, oxidative damage, antioxidants activities, ascorbic acid, glutathione

## Abstract

Extensive use of chromium (Cr) in anthropogenic activities leads to Cr toxicity in plants causing serious threat to the environment. Cr toxicity impairs plant growth, development, and metabolism. In the present study, we explored the effect of NaHS [a hydrogen sulfide; (H_2_S), donor] and silicon (Si), alone or in combination, on two chickpea (*Cicer arietinum*) varieties (Pusa 2085 and Pusa Green 112), in pot conditions under Cr stress. Cr stress increased accumulation of Cr reduction of the plasma membrane (PM) H^+^-ATPase activity and decreased in photosynthetic pigments, essential minerals, relative water contents (RWC), and enzymatic and non-enzymatic antioxidants in both the varieties. Exogenous application of NaHS and Si on plants exposed to Cr stress mitigated the effect of Cr and enhanced the physiological and biochemical parameters by reducing Cr accumulation and oxidative stress in roots and leaves. The interactive effects of NaHS and Si showed a highly significant and positive correlation with PM H^+^-ATPase activity, photosynthetic pigments, essential minerals, RWC, proline content, and enzymatic antioxidant activities (catalase, peroxidase, ascorbate peroxidase, dehydroascorbate reductase, superoxide dismutase, and monodehydroascorbate reductase). A similar trend was observed for non-enzymatic antioxidant activities (ascorbic acid, glutathione, oxidized glutathione, and dehydroascorbic acid level) in leaves while oxidative damage in roots and leaves showed a negative correlation. Exogenous application of NaHS + Si could enhance Cr stress tolerance in chickpea and field studies are warranted for assessing crop yield under Cr-affected area.

## Introduction

Naturally occurring heavy metals (HMs) are present in traces in soil, and their increased accumulation in crops poses serious health risks and threatens sustainable agriculture (Gautam et al., 2020; Jan et al., 2020). Chromium (Cr) is a toxic heavy metal with non-essential metabolic function in plants (Singh et al., 2020; Hussain et al., 2021). Compared with other environmental pollution stressors, Cr stress is prominent (Shanker et al., 2005). The distribution and translocation of Cr within plants depend on the plant species, oxidation state of Cr ions, and concentration of Cr in the growth medium (Ashraf et al., 2017). Since roots are the first to encounter Cr, the concentration of Cr is higher in roots compared to other plant organs (Singh et al., 2020). Cr has several different oxidation states, including “hexavalent” Cr^VI^ and “trivalent” Cr^III^, which are the two main stable states; however, Cr^VI^ is more phytotoxic than Cr^III^ (Ashraf et al., 2017; Shahid et al., 2017). High concentrations of Cr in soil can induce toxic effects in plants and reduce plant growth, enzyme activities, photosynthesis, nutrient absorption, and ultimately crop yield (Zaheer et al., 2020; Singh et al., 2021). Cr toxicity disrupts essential metabolic processes in plants, increases oxidative stress levels, and reduces plant growth as it induces ultrastructural modifications of the cell membrane and photosynthetic pigments, damages root cells, and disturbs nutrient uptake (Gill et al., 2015; Singh et al., 2021). In addition, the uptake of essential mineral nutrients is also altered in plants exposed to Cr stress; a significant reduction in N, P, K, and Mg in plant roots and leaves has been reported (Ulhassan et al., 2019). High concentrations of Cr overproduce reactive oxygen species (ROS) that ultimately disrupt the redox balance in plants (Anjum et al., 2017). To counteract the detrimental effects of ROS, plants have evolved with two important antioxidant defence mechanisms, namely enzymatic and non-enzymatic antioxidants systems, which scavenge and/or reduce excess ROS and improve plant performance by counteracting ROS toxicity (Gill and Tuteja, 2010; Mahmud et al., 2017).

Hydrogen sulphide (H_2_S) and silicon (Si) mitigate Cr stress, thereby improving plant growth, photosynthetic pigments, nutrients elements, and enzymatic and non-enzymatic antioxidants defence system (Ahmad et al., 2020; Kharbech et al., 2020; Yang et al., 2021). Hence, it is important to consider the effects of multiple related stresses, such as H_2_S and Si stress, on defence enzymes and antioxidant activities. This should be particularly investigated to mitigate damage induced by Cr stress in important crops such as chickpea.

Silicon (Si), the second most abundant element in the Earth’s crust after oxygen (Gong et al., 2006; Naeem et al., 2018) is an important nutrient for plants under abiotic and biotic stresses (Tripathi et al., 2014; Ranjan et al., 2021; Sharma et al., 2022). The uptake and transport of Si to the shoot depends on the chemical composition of the soil and root system (Mandlik et al., 2020). Si deposition in plant organs, particularly in chickpea, has often been attributed to stress tolerance mechanisms and has a protective role under heavy metal stress, especially that of Cr (Imtiaz et al., 2016). Several studies have suggested that Si develops tolerance for plants exposed Cr toxicity, which reduces its absorption and translocation (Ali et al., 2013; Zhang et al., 2013). In polluted soils, Si can reduce Cr toxicity, which significantly increases the physiological growth of the plants and crop yield (Kharbech et al., 2020). Si-enhanced tolerance against Cr in different species of plants, such as tomato (Alam et al., 2021), mustard (Ashfaque et al., 2017), wheat (Tripathi et al., 2015), and rice (Huda et al., 2017) has also been reported. These studies suggest the protective nature of Si accumulation against Cr toxicity. Alam et al. (2021) have reported that Si mediated alleviation of Cr toxicity in tomato plants, decreased oxidative damage, and promoted antioxidant defence system. The application of Si can enhance the antioxidant defence system of plants under Cr toxicity by enhancing the performance of several antioxidant enzymes against oxidative stress (Zeng et al., 2011), thereby enhancing plant tolerance (Adrees et al., 2015).

H_2_S acts as a signalling gaseous molecule in very low concentrations in plants where it is involved in the regulation of physiological mechanisms and enhances several plant growth processes, such as germination, root formation, as well as photosynthesis and antioxidant capacity (Liu and Lal, 2015; Li et al., 2016). Moreover, H_2_S can successfully overcome the toxicity of various metals in plants, such as Cr (Ahmad et al., 2020; Kharbech et al., 2020), As (Siddiqui et al., 2021a), Cd (Ali et al., 2014; Tian et al., 2016), Cu (Dai et al., 2016), and Pb (Chen et al., 2018). H_2_S can also have a protective role in plants under Cr toxicity by reducing oxidative stress levels (Husain et al., 2021) and improving oxidative defence machinery along with increasing chlorophyll contents and decreasing Cr uptake in different parts of plants (Alamri et al., 2020; Kushwaha and Singh, 2020b) but to the best of our knowledge, reports on the reduction of the effect of Cr toxicity by NaHS in chickpea species remain lacking.

Chickpea (*Cicer arietinum* L.) is an important pulse crop cultivated worldwide and ranks third in global production after beans and pea legume crops (Merga and Haji, 2019) with an annual grain production of 17.2 million tons (FAOSTAT, 2020). As a major source of protein, fats, vitamins, carbohydrates, and fibre (Wood and Grusak, 2007), chickpea is an appropriate source of energy and is globally used as animal fodder (Rasool et al., 2015; Ahmad et al., 2016). It is commonly grown in a wide range of climatic regions in several Asian and African countries (Varshney, 2016). Nevertheless, despite its economic and ecological importance, chickpea production has remained low due to various biotic and abiotic stresses and changing environmental conditions (Varshney, 2016; Deokar et al., 2019). Amongst abiotic stress, heavy metal stress such as Cr has been found to reduce the productivity of the chickpea cultivars by 72.49 and 86.81%, respectively (Singh et al., 2022).

Hence, the development of genetically improved high yielding varieties capable of adapting to various biotic and abiotic stresses and changing environmental conditions remains a challenge for chickpea breeders. Cr concentrations in soil affect yield production in several legume crops, such as mungbean (Jabeen et al., 2016; Singh et al., 2021), chickpea (Singh et al., 2020), and pea (Tiwari et al., 2009). Though NaHS and Si can mitigate Cr stress in many crops, their implications in ameliorating Cr^VI^ toxicity in chickpea crops remain nebulous. Hence, a better understanding of the mechanisms underlying Cr^VI^ resistance in response to NaHS and Si in plants is essential. The present study examines the synergistic effects of NaHS and Si in chickpea tolerance and resistance to individual and/or interactive Cr^VI^ toxicity in plants. For this, plant growth parameters, oxidative stress, photosynthetic pigments, essential minerals, relative water contents (RWC), and enzymatic and non-enzymatic antioxidants were evaluated. The findings of this study provide new insights into biochemical and physiological mechanisms underlying the effects of NaHS and Si in two chickpea varieties on Cr^VI^ toxicity.

## Materials and Methods

### Plant Material and Growth Conditions

Seeds of *C. arietinum* L. var. Pusa 2085 and var. Pusa Green 112 were provided by the Pulse Research Laboratory, ICAR-India Agricultural Research Institute, New Delhi (India). Healthy and uniform seeds were surface sterilized using sodium hypochlorite (0.02%) and subsequently rinsed four times using distilled water. Sterilized seeds of both varieties were sown in plastic pots filled with 6 kg acid-washed loamy soil under natural climatic conditions in the Department of Botany, Meerut College, Meerut, India. The initial chemical properties of loamy soils have been reported by Singh et al. (2020). Twelve days after germination, a half-strength Hoagland solution was applied to each pot twice with an interval of 10 days. The nutrient solution was based on that of Smith et al. (1983). After 2-week seedlings, pots were subjected to the different treatment conditions: control (without Cr), 120 μM Cr, 120 μM Cr + 2 mM NaHS, 120 μM Cr + 10 μM Si, and 120 μM Cr + 2 mM NaHS + 10 μM Si. Potassium dichromate (K_2_Cr_2_O_7_) for the Cr^VI^ (120 μM) treatment. Each treatment was repeated after an interval of 15 days. The pot experiments were arranged in a randomized design with five replicates. After 60 days of sowing (DAS), in each pot, seven seedlings were collected to measure their morpho-physiological and biochemical attributes.

### Measurement of Morpho-Physiological Attributes

The morpho-physiological attributes of the seedlings were measured, which included root length, shoot length, and fresh and dry biomass of the roots and shoots were measured. The dry biomass of the samples was measured after drying in a hot air oven at 65°C for 12 h, then at 105°C for 4 h.

### Measurement of Photosynthetic Pigments

The uppermost fully extended leaves were used to determine the chlorophyll (Chl a and b) and carotenoid contents according to the method reported by Arnon (1949). Leaf samples (0.5 g) were incubated in 10 ml of aqueous acetone (80%) followed by centrifugation at 10,000 × *g* for 10 min. The absorbance of the supernatant was determined using a spectrophotometer (UV-1800, Shimadzu, Japan) at 663 and 645 nm for estimation of chlorophylls and 480 and 510 nm for carotenoid contents.

### Detection of Stress Biomarkers (Hydrogen Peroxide, Malondialdehyde, and Electrolyte Leakage) and Proline Level

Fresh root and leaves samples (0.5 g) were homogenized and added to 5 ml of trichloroacetic acid (0.1%, w/v) and the subsequent combination was centrifuged at 10,000 × *g* for 10 min. Hydrogen peroxide (H_2_O_2_) content was detected by estimating the absorbance at 410 nm (Jana and Choudhuri, 1981). The method reported by Heath and Packer (1968) was used for estimating MDA content. The absorbance of the supernatant at 532 and 600 nm was noted, and the MDA content was calculated by subtracting the non-specific absorbance at 600 nm from the absorbance at 532 nm using an absorption coefficient of extinction of 155 mm^−1^ cm.

The assessment of electrolyte leakage followed the method described by Singh et al. (2021). Electrolyte leakage (EL%) values were calculated using the following formula given by Valentovic et al. (2006):


$$\mathrm{EL}\%=\left({\mathrm{EL}}_{1}{\mathrm{/EL}}_{2}\right)\times 100$$

where EL_1_ is the initial electrical conductivity and EL_2_ is the final electrical conductivity.

To measure the proline contents, 0.5 g fresh root and leaf samples were homogenized using 5 ml sulfosalicylic acid solution (3%) according to the method described by Bates et al. (1973). The homogenized samples were centrifuged at 5000 × *g* for 10 min. The absorbance of the homogenized samples of chickpea seedlings were estimated at 520 nm, and the content of proline were calculated using a standard curve.

### Extraction and Estimation of Relative Water Content

Relative water content was examined for chickpea leaves using the methods of Smart and Bingham (1974). The fresh weights of average leaves of the harvested plants were measured. Thereafter, the leaves were allowed to float on deionised water for 5 h. The turgid plant parts were rapidly dried by blotting, and the turgid weight was determined. The dry weight of the leaves was recorded after oven-dried at 65°C for 48 h. The RWC values were calculated using the following formula of Tousi et al. (2020):


$$\mathrm{WRC}\;\left(\%\right)=\left[\begin{array}{l} \left(\mathrm{fresh weight}-\mathrm{dry}\;\mathrm{weight}\right)/\\ \left(\mathrm{turgid weight}-\mathrm{dry}\;\mathrm{weight}\right) \end{array}\right]\times 100$$

### Extraction and Estimation of Antioxidant Enzymes

To measure enzymatic activities, 0.5 g of fresh leaf samples were ground in potassium phosphate buffer solution (pH-6.20) under pre-chilled condition. Different buffer solutions were used for each enzyme (Singh et al., 2022). Thereafter, the samples were centrifuged at 10,000 × *g* for duration of 15 min at 4°C. The supernatant was extracted and stored in microcentrifuge tubes, and used to determine enzyme activities. SOD (EC 1.15.1.1) and POD (EC 1.11.1.7) activities were examined according to the method of Zhang (1992), CAT (EC 1.11.1.6) activity was measured as described by Aebi (1984), and APX (EC 1.11.1.11) and DHAR (EC 1.8.5.1) activities were examined as described by Nakano and Asada (1981). MDHAR (EC 1.6.5.4) activity was evaluated according to the method described by Hossain et al. (1984). The modification in absorbance was measured at a wavelength of 340 nm for 3 min. The activities of all enzymes were indicated as UA mg g^−1^ protein using an Ultraviolet–Visible spectrophotometer (Model-640D, Beckman, United States).

### Extraction and Determination of Non-enzymatic Antioxidants

Fresh leaf samples (500 mg) were homogenized using 3 ml of ice-cold 5% meta-phosphoric acid, which contains 1 mM EDTA. Thereafter, the samples were centrifuged at 1,1500 × *g* for 15 min. Reduced and total ascorbic acid (AsA) contents were measured at 265 nm in 0.1 M K-phosphate buffer (pH-7.0) using 1.0 unit of ascorbate oxidase (AO) as per the methods described by Mostofa and Fujita (2013). The dehydroascorbic acid (DHA) content was calculated by subtracting the reduced AsA obtained from the total amount of AsA present. Reduction in glutathione (GSH) and glutathione disulphide (GSSG) levels was detected using the method described by Griffith (1980). The reduced GSH content was determined by subtracting the value of GSSG from the total GSH level.

### Measurement of Plasma Membrane H^+^-ATPase Activity

Plasma membrane (PM) H^+^-ATPase activity in the fresh leaf samples of chickpea plants based on the detailed protocol of Hejl and Koster (2004) and with minor modifications by Singh et al. (2022). PM H^+^-ATPase activity was measured by quantifying the release of inorganic phosphate at an absorbance of 700 nm using a spectrophotometer (BRS, Brussels, Belgium).

### Detection of Nutrient Elements and Cr Uptake

Root and leaf samples were harvested from 60 day-old chickpea varieties, washed thrice with tap water, and then rinsed twice with deionized water to remove other adhering nutrients. Thereafter, the root and leaf samples were dried in a hot air oven at 65°C for 12 h, and then at 105°C for 4 h, and the dried samples were ground into powder. Root and leaf samples (0.5 g) were digested with a mix of 1 ml of HClO_4_ + 5 ml of HNO_3_. The resulting solution was diluted to 25 ml using 2% HNO_3_ and then filtered. The concentrations of Ca, K, Mg, and Cr in the filtrate were measured using an atomic absorption spectrometer-AAS (ZEEnit 700P, Analytik Jena, Germany). Phosphorus (P) and nitrogen (N) levels were measured according to the methodology of Murphy and Riley (1962) and Singh et al. (2020), respectively.

### Statistical Analysis

Statistical analysis of the data was performed using one-way ANOVA using SAS software version 9.4 (United States). Data were expressed as mean ± SE (*n* = 5) and significant differences between mean values were identified based on the least significant difference test at *p* < 0.05. The Pearson correlation coefficient was also calculated between the measured variables for different parameters of chickpea plants.

## Results

### Effects of NaHS and Si on the Plant Growth Characteristics Under Cr^VI^ Toxicity

**Figure 1:**
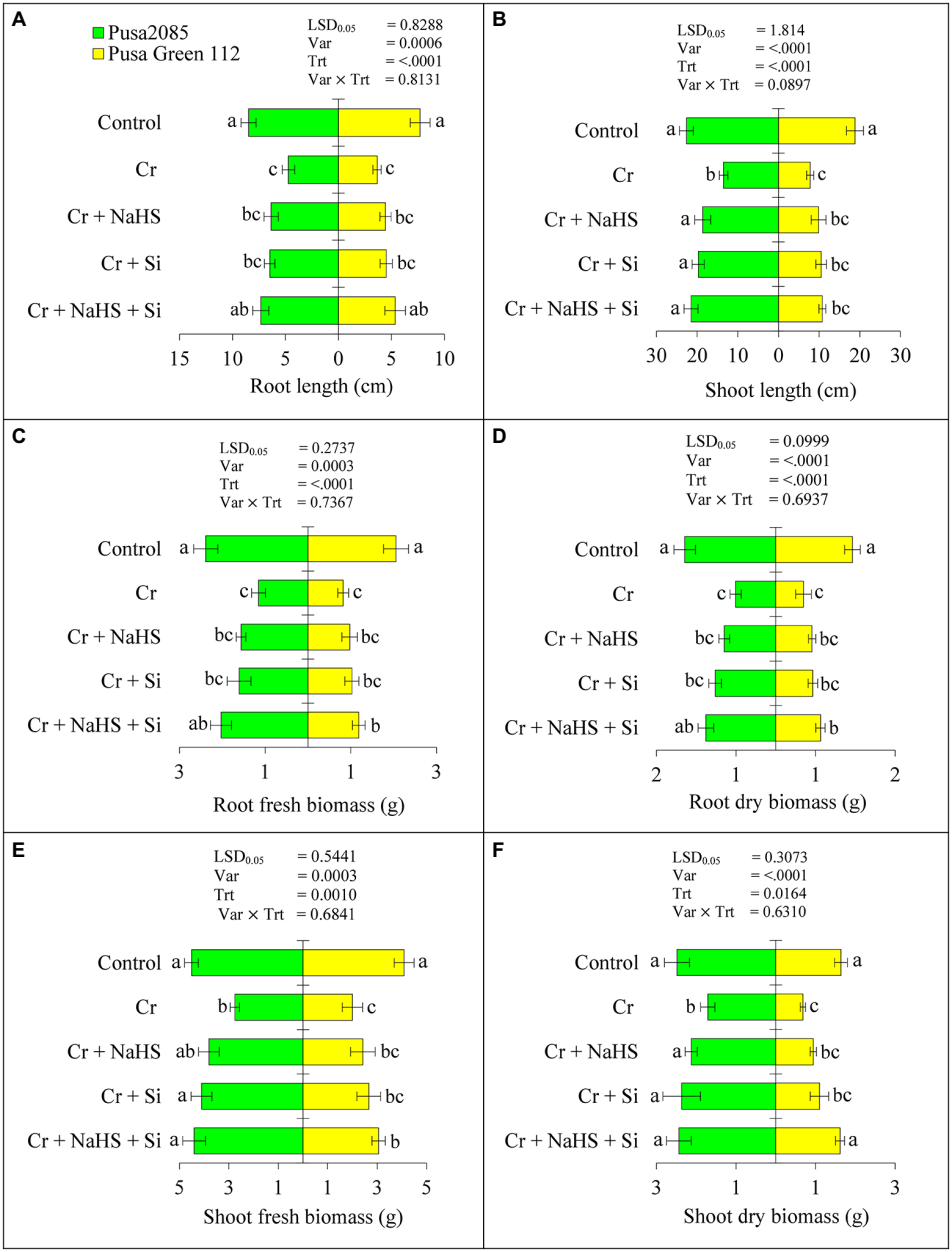
Effect of NaHS, Si, and NaHS + Si treatments on **(A)** root length (RL)-root length (cm), **(B)** shoot length (SL)-shoot length (cm), **(C)** RFB-root fresh biomass (g), **(D)** RDW-root dry biomass (g), **(E)** SFW-shoot fresh biomass (g), and **(F)** SDW-shoot dry biomass (g) of both chickpea plants grown during Cr^VI^ stress. Data are presented as mean ± SE (*n* = 5), and different bar letters are indicated statistically differences (*p* < 0.05) as tested by the least significant difference test.

Results related to growth characteristics such as root and shoot lengths and fresh and dry biomass of treated and un-treated plants of both chickpea varieties (Pusa 2085 and Pusa Green 112) are presented in Figure 1. Cr^VI^ treatment (120 μM) significantly reduced the root and shoot length by 44.43 and 40.35% in Pusa 2085 and 52.67 and 58.54% in Pusa Green 112, respectively. Furthermore, the Cr^VI^ stress conditions significantly reduced the fresh biomass of root and shoot of the Pusa 2085 variety to 51.61 and 38.94% and 60.06 and 51.14% in Pusa Green 112, respectively, when compared with the controls. However, exogenous application of NaHS with Cr^VI^ stress enhanced the root and shoot length by 25.80 and 27.57% in Pusa 2085 and 17.94 and 20.96% in Pusa Green 112, respectively, when compared with the controls (Figure 1). The fresh biomass of root and shoot was enhanced by 26.12 and 27.58% in Pusa 2085 and 15.52 and 17.45% in Pusa Green 112, respectively, as compared with the controls (Figure 1). Moreover, exogenous application of Si with Cr significantly enhanced the root and shoot lengths and fresh and dry biomass (Figure 1). Compared with Cr^VI^ treated chickpea plants, combined application of NaHS + Si with Cr^VI^ increased the root and shoot length by 35.68 and 37.22%, respectively, in Pusa 2085 and 31.96 and 27.74% in Pusa Green 112. Similarly, fresh biomass of root and shoot was increased by 43.12 and 37.41% and dry biomass by 42.47 and 29.73%, respectively, in Pusa 2085, while the fresh biomass of Pusa Green 112 root and shoot was increased by 30.83 and 34.63% and dry biomass was 34.40 and 29.17%, respectively. Notably, the separate application of NaHS and Si mitigated the detrimental effects of Cr^VI^ on growth-related parameters, such as root and shoot lengths as well as fresh and dry biomass of roots and shoots in both chickpea varieties, Pusa 2085 and Pusa Green 112. A remarkable interaction effect was observed with the combined exogenous application of NaHS + Si + Cr^VI^ for all growth characteristics. Moreover, NaHS + Si enhancement effects were more prominent in Pusa 2085 than in Pusa Green 112.

### Effects of NaHS and Si on the Photosynthesis Pigments Under Cr^VI^ Toxicity

**Figure 2:**
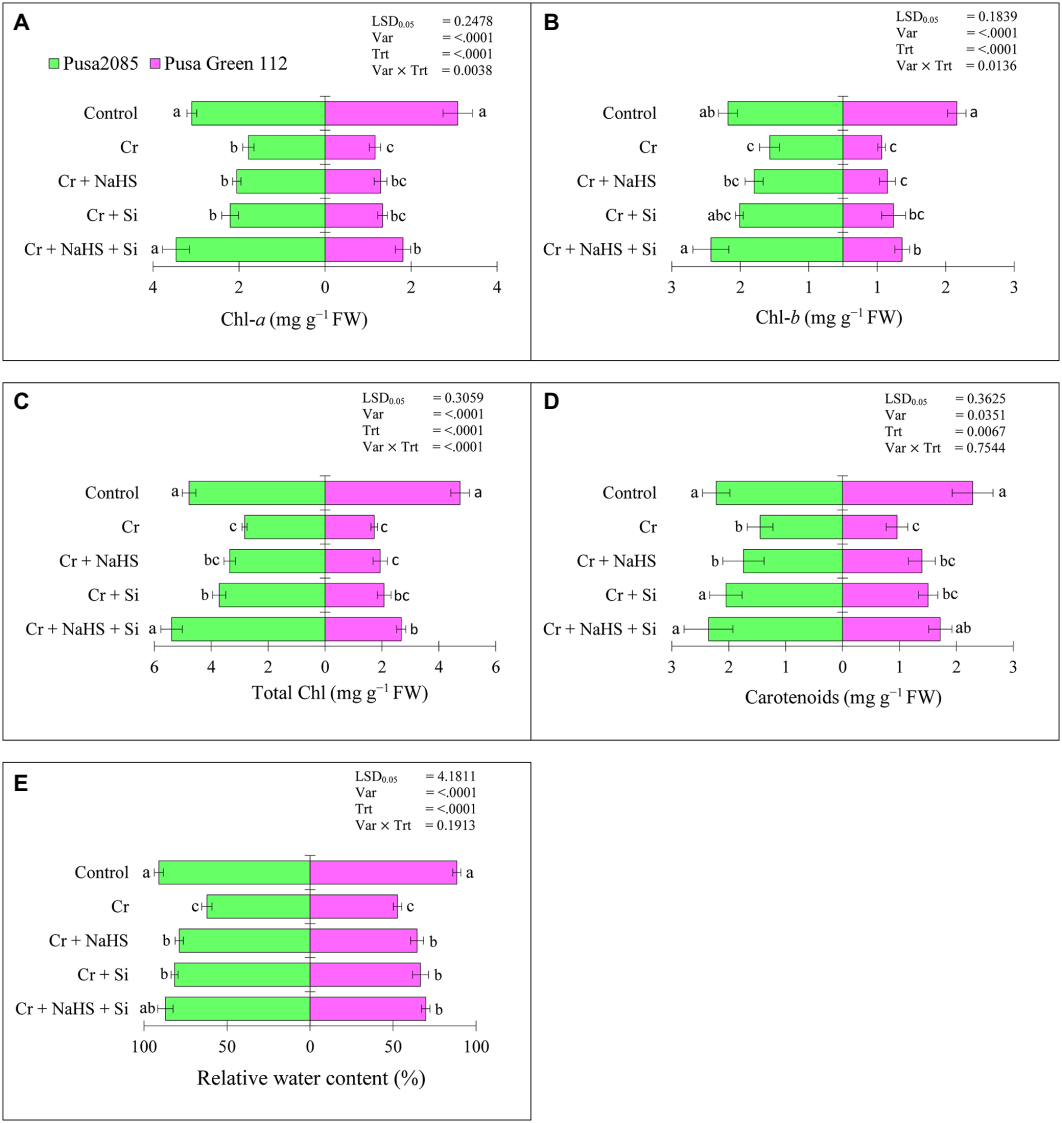
Effect of NaHS, Si, and NaHS + Si treatments on **(A)** chlorophyll-*a*, **(B)** chlorophyll-*b*, **(C)** total chlorophyll, **(D)** carotenoids, and **(E)** relative water contents in leaves of both chickpea plants grown during Cr^VI^ stress. Data are presented as mean ± SE (*n* = 5) and different bar letters are indicated statistically differences (*p* < 0.05) as tested by the least significant difference test.

Chromium (Cr)^VI^ stress decreased Chl *a* (42.44 and 62.33%), Chl *b* (36.24 and 66.03%), total Chl (40.79 and 63.62%), and carotenoid (34.69 and 58.16%) contents in Pusa 2085 and Pusa Green 112, respectively, compared to the control plants (Figures 2A–D). The individual application of NaHS with Cr^VI^ stress enhanced Chl *a* (13.09 and 10%), Chl *b* (17.43 and 12.95%), total Chl (15.53 and 10.99%), and carotenoids (16.78 and 21.54%) in the Pusa 2085 and Pusa Green 112, whereas Si application also enhanced Chl *a* (19.19 and 13.12%), Chl *b* (29.19 and 23.70%), total Chl (23.94 and 16.89%), and carotenoids (29.25 and 36.43%), compared to those in the Cr^VI^ treated plants (Figures 2A–D). Furthermore, the combined application of NaHS + Si with Cr^VI^ stress enhanced Chl *a* (48.56 and 35.76%), Chl *b* (44.47 and 34.76%), total Chl (47.56 and 35.75%), and carotenoids (38.45 and 44.36%) in Pusa 2085 and Pusa Green 112 compared to Cr^VI^ treated plants. Compared to the separate application of NaHS and Si, the negative effects of Cr^VI^ on photosynthetic pigments were substantially reduced by the combined application of NaHS + Si in plants under Cr^VI^ stress. Moreover, the results show that NaHS + Si application improved the photosynthesis pigments, which was more obvious in Pusa 2085 under Cr^VI^ stress than in Pusa Green 112.

### Effects of NaHS and Si on MDA, H_2_O_2_, EL, and Proline Levels Under Cr^VI^ Toxicity

**Figure 3:**
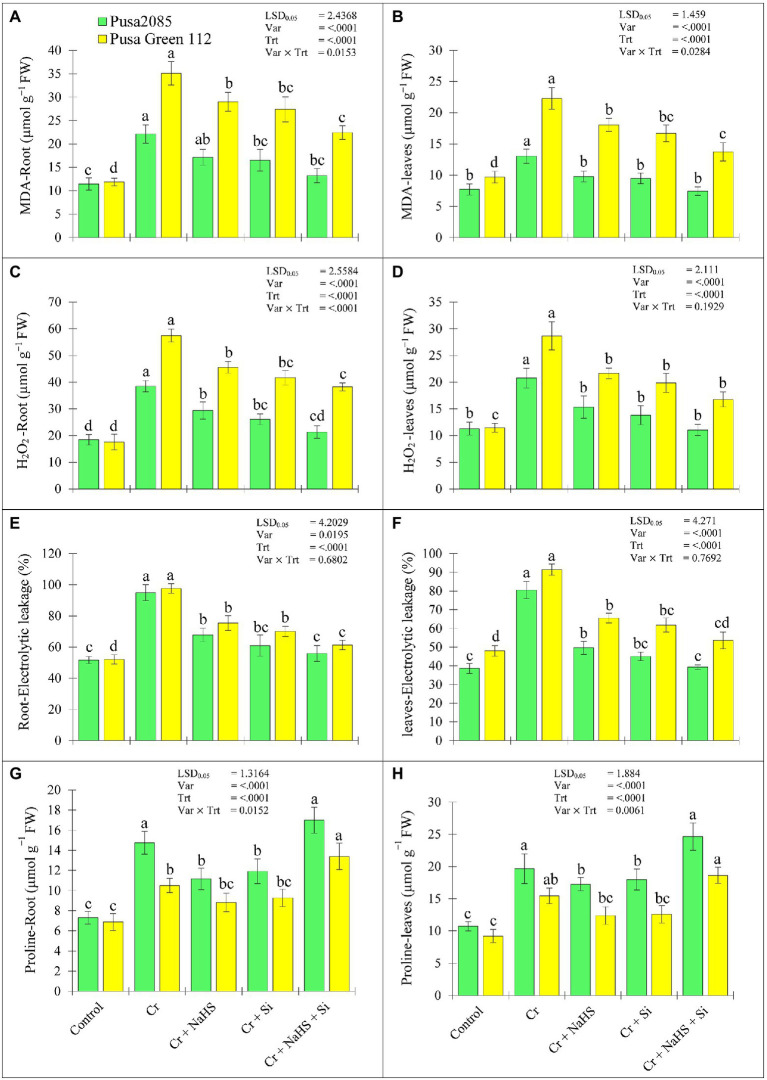
Effect of NaHS, Si, and NaHS + Si treatments on **(A, B)** MDA—malondialdehyde, **(C, D)** H_2_O_2_—hydrogen peroxide, **(E, F)** EL%—electrolyte leakage, and **(G, H)** proline amount in roots and leaves of both chickpea plants grown during Cr^VI^ stress. Data are presented as mean ± SE (*n* = 5) and different bar letters are indicated statistically differences (*p* < 0.05) as tested by the least significant difference test.

Increasing percentages of MDA, H_2_O_2_, and EL as well as proline content in roots and leaves were observed in both the chickpea varieties under Cr^VI^ stress (Figure 3). The NaHS treatment reduced MDA, H_2_O_2_, and EL content in roots of Pusa 2085 by 22.53, 23.72 and 27.12%, respectively and leaves by 24.93, 26.13 and 30.87%, respectively, in Cr^VI^ treated chickpea plants (Figures 3A–F). Similar results were observed in the roots and leaves of Pusa Green 112 variety (Figures 3A–F). In addition, compared to the Cr^VI^ treated plants, the exogenous application of Si in plants under Cr^VI^ stress significantly reduced the MDA, H_2_O_2_, and EL contents in the Pusa 2085 roots by 25.24, 32.22 and 33.97%, respectively, whereas in the leaves of Pusa 2085, there was a decline of 27.20, 33.51 and 35.44%, respectively. Similarly, reduced the MDA, H_2_O_2_, and EL contents were observed in the Pusa Green 112 roots by 21.99, 27.43, and 27.57%, respectively, while the leaves were declined by 25.11, 30.67, and 29.64%, respectively. Moreover, NaHS + Si in Pusa 2085 variety plants under Cr^VI^ stress significantly reduced the amounts of MDA (by 40.14 and 42.98%), H_2_O_2_ (by 44.64 and 46.75%), and EL (by 39.03 and 41.20%) in the roots and leaves, respectively, in Cr^VI^ treated chickpea plants. Similarly, the content of MDA in the roots and leaves of Pusa Green 112 was declined by 36.15 and 38.44%, of H_2_O_2_ by 33.47 and 41.45%, and of EL by 36.34 and 45.87%, respectively. The combined supplementation of NaHS and Si significantly declined the negative effects of ROS and lipid peroxidation in both varieties of chickpea but the negative effect was more decline in Pusa 2085 as compared to Pusa Green 112 variety. Interestingly, our results showed that Pusa 2085 was overall more tolerant than Pusa Green 112.

In Cr^VI^ treated chickpea plants, NaHS treatment under Cr^VI^ stress increased proline levels in root and leaves of Pusa 2085 by 34.55 and 37.95% respectively, and the Pusa Green 112 by 22.02 and 25.59%, respectively. Meanwhile, Si treatment increased the proline levels in the root and leaves of Pusa 2085 variety (by 38.71 and 40.44%, respectively) and Pusa Green 112 (by 25.94 and 26.77%, respectively) compared to Cr^VI^ treated chickpea plants. Moreover, NaHS + Si treatment further enhanced the amount of proline in root and leaves of Pusa 2085 (by 61.56 and 62.61%, respectively) and Pusa Green 112 (by 48.75 and 50.56%, respectively; Figures 3G,H). The results show that NaHS + Si application enhanced the proline levels in the roots and leaves of both chickpea varieties, but was more obvious in Pusa 2085 than Pusa Green 112 under Cr^VI^ stress.

### Effects of NaHS and Si on Relative Water Contents Under Cr^VI^ Toxicity

The RWCs in leaves of both varieties were significantly decreased under Cr^VI^ treated chickpea plants (Figure 2E) and enhanced following separate or combined treatment with NaHS and Si (Figure 2E). NaHS alone increased RWC by 35% in Pusa 2085 leaves and 27% in Pusa green 112 under Cr stress in Cr^VI^ treated chickpea plants (Figure 2E). In contrast, Si alone increased RWC by 36% in Pusa 2085 leaves and 31% in Pusa Green 112 compared to Cr treated plants. Furthermore, NaHS + Si treatment enhanced RWC by 30.71 and 24% in Pusa 2085 and Pusa Green 112, respectively in Cr^VI^ treated chickpea plants. The results indicated that combined supplementation of NaHS and Si was more effective in Cr^VI^ stress reduction of chickpea plants by increasing RWC.

### Effects of NaHS and Si on the Enzymatic of Antioxidants Under Cr^VI^ Toxicity

**Figure 4:**
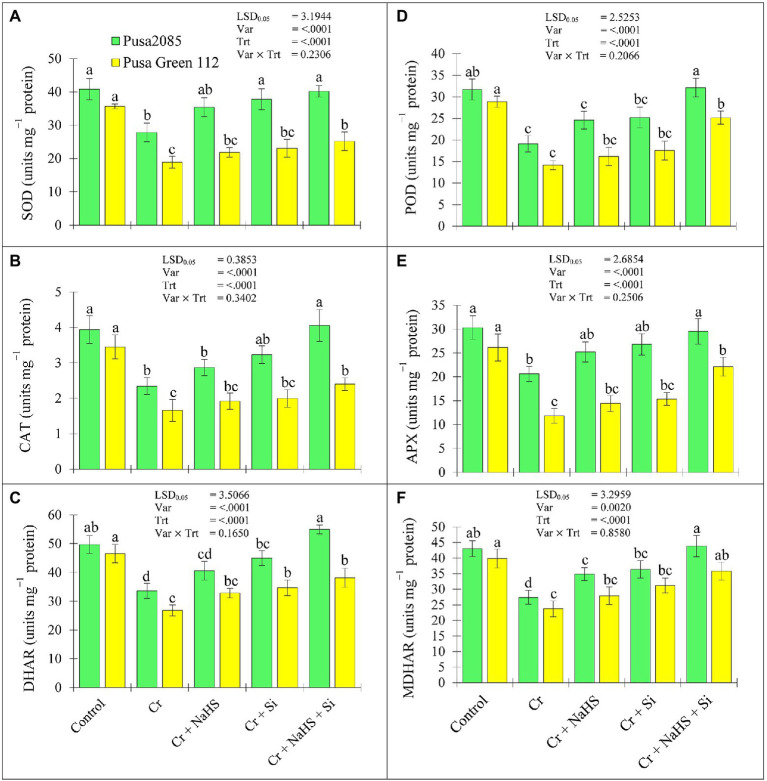
Effect of NaHS, Si, and NaHS + Si treatments enzymatic activities of **(A)** SOD-superoxide dismutase, **(B)** CAT-catalase, **(C)** DHAR-dehydroascorbate reductase, **(D)** POD-peroxidase, **(E)** APX-ascorbate peroxidase, **(F)** MDHAR-monodehydroascorbate reductase in leaves of both chickpea plants grown during Cr^VI^ stress. Data are presented as mean ± SE (*n* = 5) and different bar letters are indicated statistically differences (*p* < 0.05) as tested by the least significant difference test.

The activity of all enzymatic antioxidants was significantly reduced in the leaves of both chickpea varieties grown in Cr^VI^ treated soil (Figure 4). The individual application of NaHS in plants under Cr^VI^ stress enhanced the content of enzymatic antioxidants, including SOD, CAT, DHAR, POD, APX, and MDHAR in the leaves of the Pusa 2085 variety (by 21.36, 18.30, 17.35, 22.29, 18.22, and 21.43%, respectively) and Pusa Green 112 variety (by 13.61, 13.46, 18.30, 12.40, 18.05, and 15.01%, respectively), compared with those in the Cr^VI^ treated plants. In addition, Si treatment increased the contents of SOD, CAT, DHAR, POD, APX, and MDHAR in the Pusa 2085 leaves (by 26.35, 27.41, 25.38, 24.13, 23.10, and 24.75%, respectively) and Pusa Green 112 (by 18.30, 16.83, 22.64, 19.30, 22.91, and 23.92%, respectively) in Cr^VI^ treated chickpea plants. Furthermore, the combined exogenous application of NaHS + Si under Cr^VI^ stress enhanced the activities of SOD, CAT, DHAR, POD, APX, and MDHAR in Pusa 2085 (by 30.71, 42.20, 38.96, 40.51, 30.17, and 37.52%, respectively) and Pusa Green 112 (by 25.07, 30.89, 29.74, 29.86, 26.60, and 33.71%, respectively; Figure 4). Overall Si + NaHS treatment showed a maximum significant increase in all enzymatic activities under Cr^VI^ stress in both chickpea plants, but all enzymatic activities were higher in Pusa 2085 (tolerance variety) than Pusa Green 112 (sensitive variety; Figure 4), which indicated that the Pusa 2085 can more strongly tolerate the toxic effects of Cr^VI^.

### Effects of NaHS and Si on the Non-enzymatic Activities Under Cr^VI^ Toxicity

**Figure 5:**
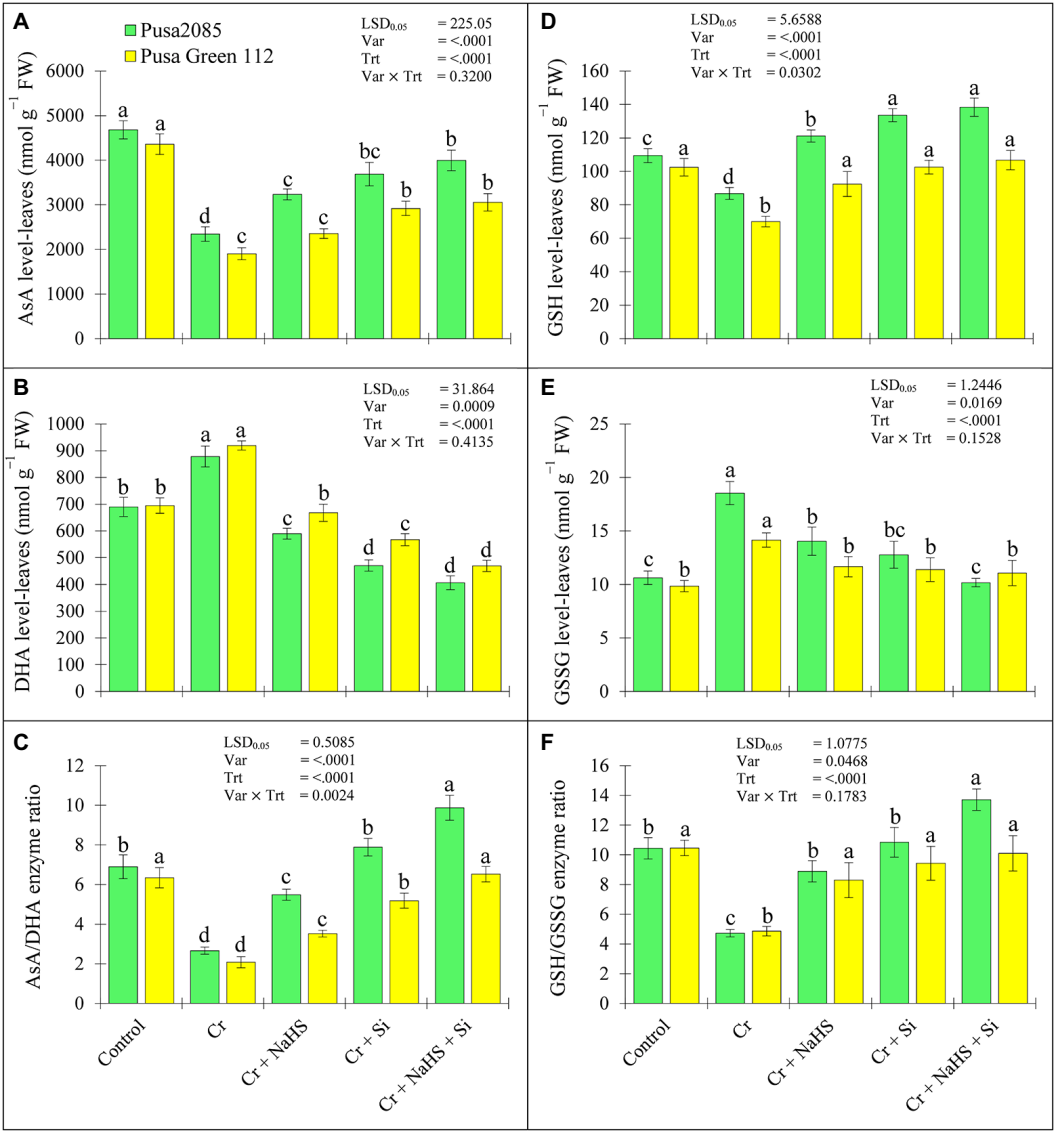
Effect of NaHS, Si, and NaHS+Si treatments non-enzymatic activities of **(A)** AsA-ascorbic acid, **(B)** DHA-dehydroascorbic acid, **(C)** AsA/DHA enzyme ratio, **(D)** GSH-glutathione, **(E)** GSSG-oxidized glutathione, and **(F)** GSH/GSSG enzyme ratio in leaves of both chickpea plants grown during Cr^VI^ stress. Data are presented as mean ± SE (*n* = 5) and different bar letters are indicated statistically differences (*p* < 0.05) as tested by the least significant difference test.

The results of ascorbate acid (AsA), dehydroascorbic acid (DHA), reduced glutathione (GSH) and oxidized glutathione (GSSG), AsA/DHA, and GSH/GSSG enzyme ratios are presented in Figure 5. The Cr^VI^ stress caused a significant decline in AsA and GSH levels and enhanced the DHA and GSSG levels in leaves of both chickpea varieties resulting in a significant decline in the ASA/DHA and GSH/GSSG enzyme ratios. In the control plants, the Cr^VI^ stress decreased AsA levels by 49.93 and 56.35%, respectively, and increased DHA levels by 21.45 and 24.43%, respectively, in the leaves of both varieties of Pusa 2085 and Pusa Green 112 (Figure 5). Similarly, GSH levels in leaves of both chickpea varieties declined by 20.65 and 31.69%, respectively, while GSSG levels increased by 42.68 and 32.14%, respectively. However, AsA/DHA and GSH/GSSG enzyme ratios were significantly declined under Cr^VI^ treated chickpea plants (Figures 5C,F). The results showed that application of NaHS and Si, alone or in combination, increased the levels AsA and GSH, and improved the ratios of AsA/DHA and GSH/GSSG in Cr^VI^ treated both chickpea plants, whereas DHA and GSSG levels were decreased (Figure 5). In the control plants, the combined treatment of NaHS + Si shown a maximum improvement of AsA and GSH levels in Pusa 2085 leaves by 41.35 and 37.26%, while that in Pusa Green 112 improved by 37.72 and 34.42%, respectively. NaHS + Si treatment decreased DHA and GSSG levels in Pusa 2085 by 53.72 and 45.14%, respectively and Pusa Green 112 by 48.49 and 23.70%, respectively as compared to the Cr^VI^ treated chickpea plants (Figures 5B,E). In addition, externally applied NaHS and Si, alone and or in combination, resulted in a significant increase in the levels of AsA and GSH with lower levels of DHA and GSSG resulting in an increase in the AsA/DHA and GSH/GSSG enzyme ratios by promoting the antioxidant capacity of Cr^VI^-treated chickpea plants. These results suggest that increased AsA and GSH levels and decreased DHA levels contributed to the decrease of Cr^VI^ stress in chickpea plants by promoting the antioxidant capacity of Cr^VI^ treated chickpea plants.

### Effects of NaHS and Si on Plasma Membrane H^+^-ATPase Activity Under Cr^VI^ Toxicity

**Figure 6:**
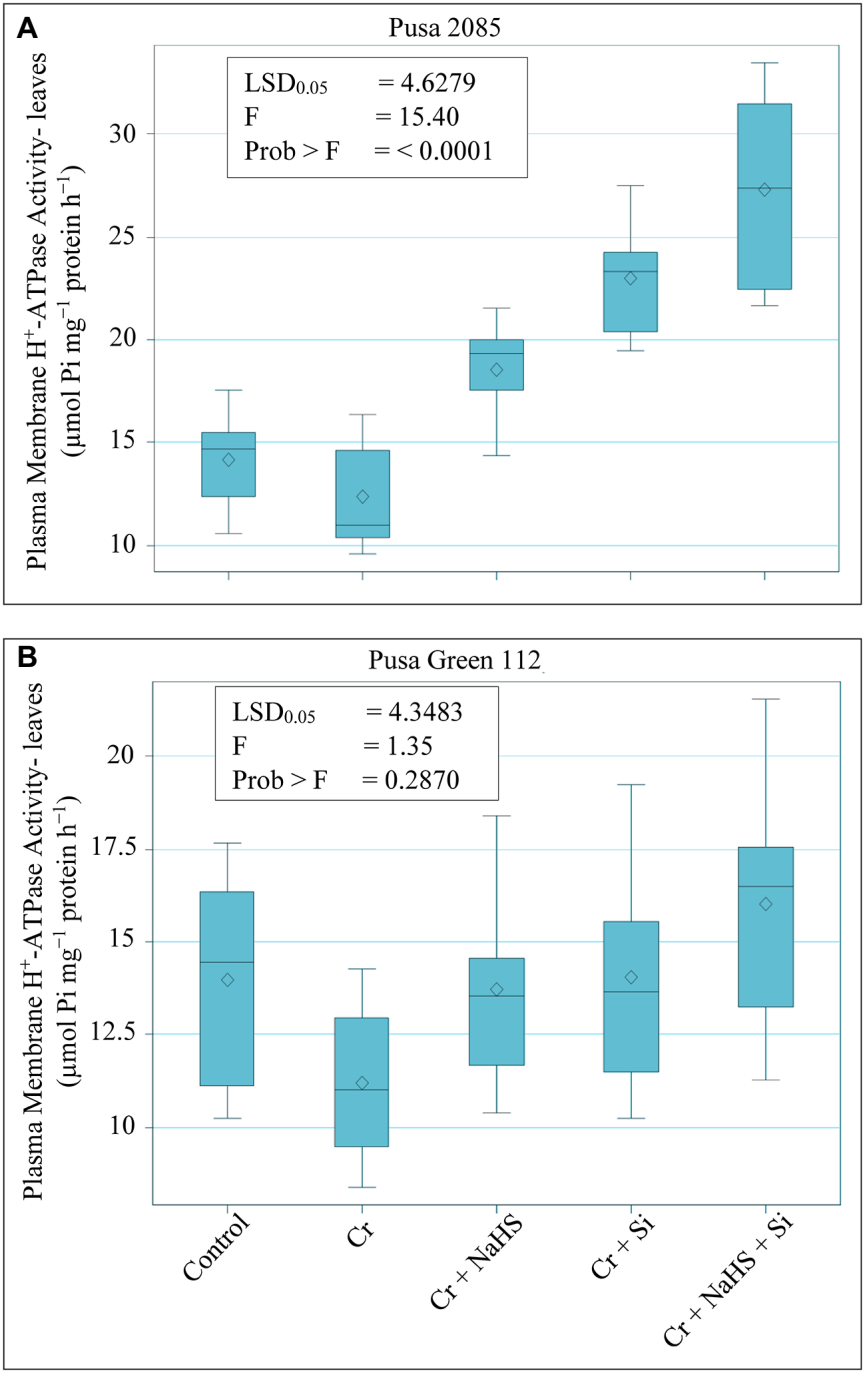
Effect of NaHS, Si, and NaHS + Si treatments on plasma membrane H^+^-ATPase activity in leaves of both chickpea plants **(A)** Pusa 2085, and **(B)** Pusa Green 112 grown during Cr^VI^ stress. Data are presented as mean ± SE (*n* = 5) and different bar letters are indicated statistically differences (*p* < 0.05) as tested by the least significant difference test.

The results related to PM H^+^-ATPase activity in leaves of both chickpea varieties are depicted in Figure 6. Cr^VI^ treatment (120 μM) declined PM H^+^-ATPase activity by 12.31% in Pusa 2085 leaves and 19.77% in Pusa Green 112 leaves as compared to the untreated chickpea plants. However, NaHS treatment increased PM H^+^-ATPase activity in the leaves of Pusa 2085 and Pusa Green 112 by 33.23 and 18.23%, respectively, whereas the Si increased PM H^+^-ATPase activity by 46.04 and 20.17%, respectively, compared to Cr^VI^ treated chickpea plants (Figure 6). Furthermore, NaHS + Si treatment more prominently reduced stress by increasing PM H^+^-ATPase activity compared to Cr^VI^ treated chickpea plants by 54.54 and 30.01% in leaves of Pusa 2085 and Pusa Green 112, respectively. Generally, enhanced effects Si and NaHS treatments, alone or in combination, were found on PM H^+^-ATPase activity in leaves of both chickpea varieties under Cr^VI^ stress. Our results showed that the H^+^-ATPase activity in leaves of Pusa 2085 (tolerant variety) was higher than that of Pusa Green 112 (sensitive variety).

### Effects of NaHS and Si on Nutrient Elements Under Cr^VI^ Toxicity

The results related to mineral elements in roots and leaves of both chickpea varieties are listed in Supplementary Table S1. Mineral elements such as N, P, K, Ca, and Mg showed significantly declined in the roots and leaves of both chickpea varieties under Cr^VI^ stress as compared with respective untreated chickpea plants. In Cr^VI^ treated chickpea plants, the individual application of NaHS enhanced the accumulation of N, P, K, Ca, and Mg elements in the roots and leaves of both the chickpea varieties (Supplementary Table S1). Similarly, Si increased rates of mineral elements such as N, P, K, Ca, and Mg in the roots and leaves of both chickpea varieties in Cr^VI^ treated chickpea plants (Supplementary Table S1). Furthermore, the combined exogenous application of NaHS + Si under Cr^VI^ stress also enhanced the accumulation of N, P, K, Ca, and Mg in the roots and leaves of both chickpea varieties (Supplementary Table S1). Treatments of NaHS and Si, when supplied separately, mitigated the effects of Cr on all mineral accumulation measured in the chickpea plants. A remarkable interactive effect of the combined application of NaHS and Si in plants under Cr stress was also observed for all nutrient elements (Supplementary Table S1). Higher accumulation of these elements was observed in Pusa 2085 (tolerant variety) than in Pusa Green 112 (sensitive variety).

### NaHS and Si Suppress Cr Uptake and Accumulation to Overcome Cr^VI^ Toxicity

**Figure 7:**
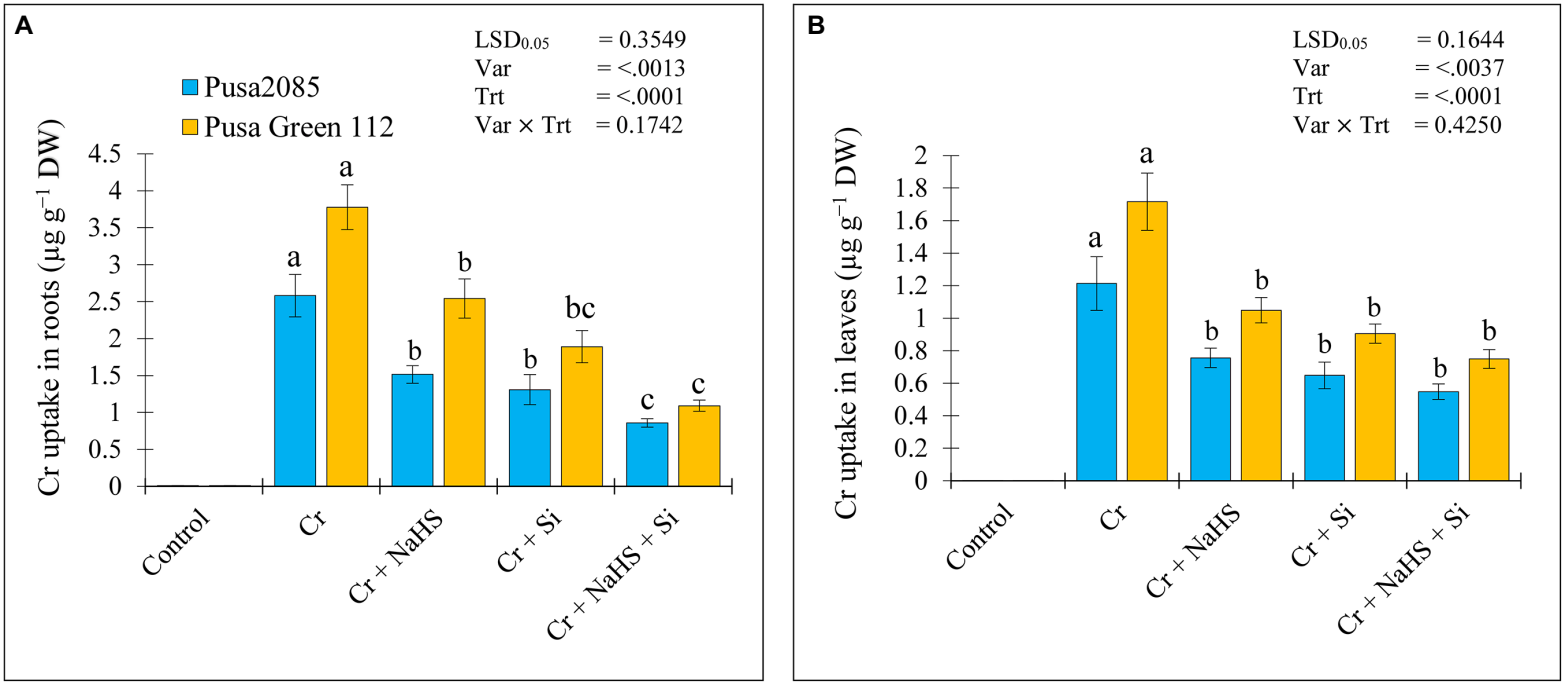
Effect of NaHS, Si, and NaHS + Si treatments on Cr accumulation in roots **(A)** and leaves **(B)** of both chickpea plants grown during Cr^VI^ stress. Data are presented as mean ± SE (*n* = 5) and different bar letters are indicated statistically differences (*p* < 0.05) as tested by the least significant difference test.

Under Cr^VI^ stress, the roots and leaves of Pusa 2085 plants showed Cr accumulation of 2.58 and 1.21 μg g^−1^ dry weight in roots and leaves, respectively, whereas roots and leaves of Pusa Green 112 showed Cr accumulation of 3.78 and 1.71 μg g^−1^ dry weight, respectively. Cr content in the roots and leaves of both the chickpea varieties increased under Cr^VI^ stress, and was higher than that in leaves of both chickpea varieties (Figure 7). NaHS treatment reduced the Cr accumulation in roots and leaves of Cr^VI^-stressed Pusa 2085 by 1.52 and 0.75 μg g^−1^ dry weight, respectively and of Pusa Green 112 by 2.54 and 1.04 μg g^−1^ dry weight, respectively. Si treatment also showed a reduction in Cr accumulation, as shown in Figure 7. The exogenous treatment of NaHS + Si in plants under Cr^VI^ stress further declined the Cr accumulation in the roots and leaves of Pusa 2085 by 0.85 and 0.54 μg g^−1^ dry weight, respectively, and of roots and leaves in Pusa Green 112 by 1.09 and 0.75 μg g^−1^ dry weight, respectively (Figure 7). These results indicated that exogenous treatments of NaHS, Si, and NaHS + Si reduced the Cr content in roots and leaves of both chickpea varieties under Cr^VI^ stress.

### Correlation Analysis Between Growth, Physiological and Biochemical Parameters, and Cr Accumulation in Chickpea Plants

**Figure 8:**
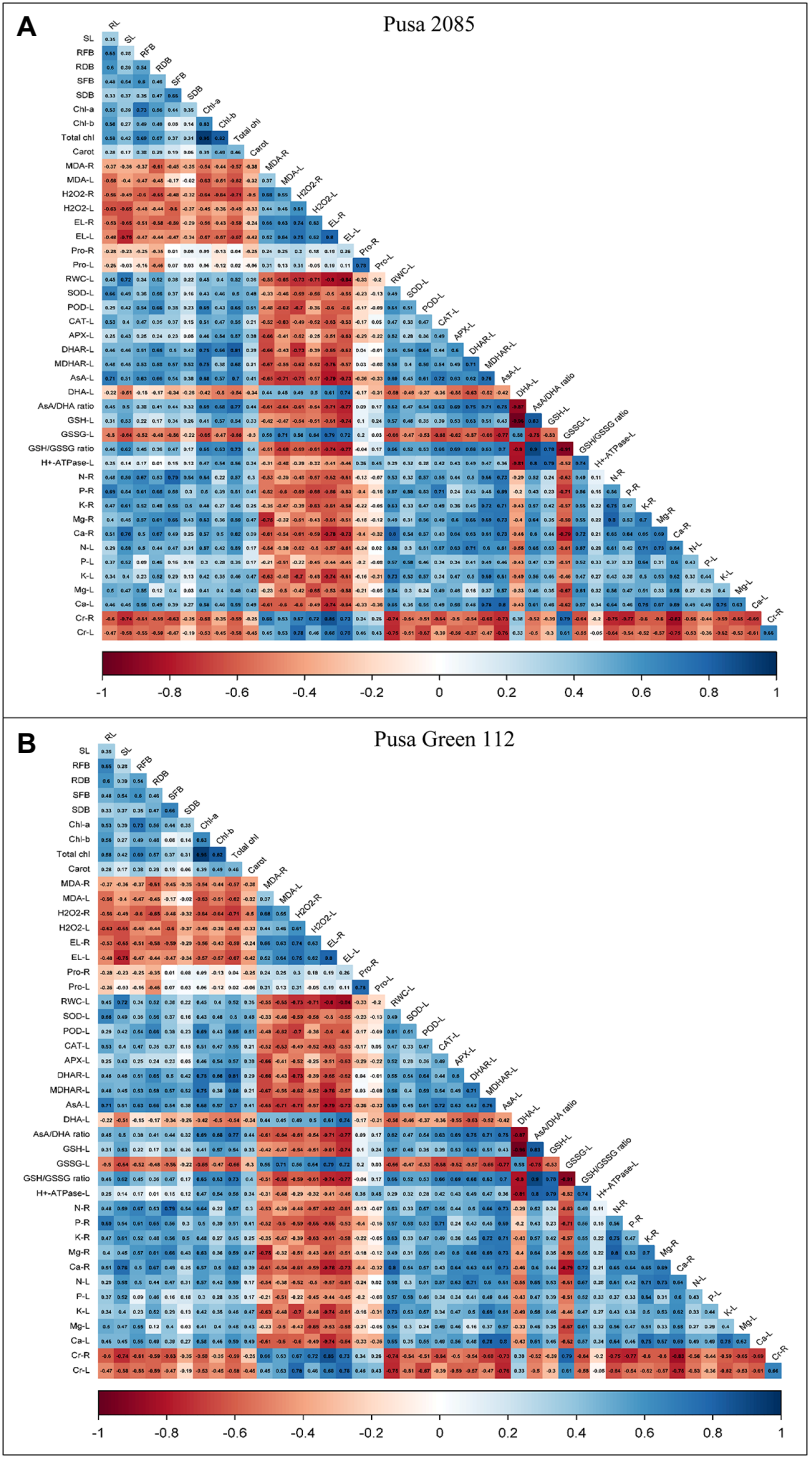
Correlation between growth, physiological and biochemical parameters, NaHS, Si alone or in the interactive treatment of NaHS + Si with Cr uptake in different parts of both chickpea varieties **(A)** Pusa 2085 and **(B)** Pusa Green 112. RL, Root length; SL, Shoot length; RFB, Root fresh biomass; SFB, Shoot fresh biomass; RDB, Root dry biomass; SDB, Shoot dry biomass; Chl-*a*, Chlorophyll-*a*; Chl-*b*, Chlorophyll-*b*; Carot, Total chlorophyll, Carotenoid contents; RWC, relative water contents; H_2_O_2_-R, H_2_O_2_ level in roots; H_2_O_2_-L, H_2_O_2_ level in leaves; MDA-R, MDA level in roots; MDA-L, MDA level in leaves; EL-R, Electrolyte leakage in roots; EL-L, Electrolyte leakage in leaves; Pro-R, Proline level in roots; Pro-L, Proline level in leaves; SOD-L, SOD enzyme in leaves; POD-L, POD enzyme in leaves; CAT-L, CAT enzyme in leaves; APX-L, APX enzyme in leaves; DHAR-L, DHAR enzyme in leaves; MDHAR-L, MDHAR enzyme in leaves; AsA-L, AsA level in leaves; DHA-L, DHA level in leaves; GSH-L, GSH level in leaves; GSSG-L, GSSG level in leaves; AsA/DHA ratio, AsA/DHA level in leaves; GSH/GSSG ratio, GHS/GSSG level in leaves; H^+^-ATPase-L, H^+^-ATPase activity in leaves; N-R, Nitrogen content in roots; N-L, Nitrogen content in leaves; P-L, Phosphorus content in leaves; P-R, Phosphorus content in roots; K-R, Potassium content in roots; K-L, Potassium content in leaves; Ca-R, Calcium content in roots; Ca-L, Calcium content in leaves; Mg-R, Magnesium content in roots; Mg-L, Magnesium content in leaves; Cr-R, Chromium contents in roots; and Cr-L, Chromium contents in leaves.

A person’s correlation was constructed to assess the relationship between the various growth, physiological, and biochemical parameters and Cr accumulation in different parts of plants (Figures 8A,B). Cr accumulation in the roots and leaves of both varieties was positively correlated with oxidative damage (MDA, H_2_O_2_, EL, and proline contents in the respective organs). However, they showed negative correlation with H^+^-ATPase activity, photosynthetic pigments, RWC, and enzymatic (CAT, POD, APX, DHAR, SOD, and MDHAR) and non-enzymatic (AsA and GHS) antioxidants in the leaves as well as with essential nutrients in the roots and leaves. In contrast, positive correlation was observed between GSSG and proline levels in roots and leaves (Figure 8). The individual and combined treatments of NaHS and Si showed a highly positive correlation with PM H^+^-ATPase activity, photosynthetic pigments, all enzymatic antioxidants activities, non-enzymatic antioxidant activities (AsA and GSH levels), proline contents, RWC, essential minerals, and negative correlation between DHA and GSSG levels in leaves and oxidative damage (MDA, EL, and H_2_O_2_) in roots and leaves (Figure 8). These results suggest that individual as well as combined treatment of NaHS and Si improved plant growth and decreased Cr concentration.

## Discussion

Chromium is a miscellaneous industrial element that accumulates in the environment, thereby polluting the agricultural ecosystem. The presence of Cr can also have harmful effects on plants and animals. Several techniques have been used to enhance plant growth and mitigate these toxic effects. For example, H_2_S and Si are known to promote plant growth and development and play an effective role in plant tolerance mechanisms against various heavy metal stresses (Mahmud et al., 2017; Kaya et al., 2020a; Arif et al., 2021).

Chromium can disrupt normal metabolic processes in plants (Shahid et al., 2017; Singh et al., 2021). The present study showed that Cr stress reduces the growth and biomass production in two chickpea varieties. Under Cr^VI^ stress, the reduction in fresh biomass in both chickpea varieties tested is attributed to increased Cr uptake. Cr exposure decreases overall plant growth (especially length of roots and shoots) and biomass production in different crop species, such as oilseed rape (Gill et al., 2015; Ulhassan et al., 2019), mungbean (Singh et al., 2021), rice (Ma et al., 2016), and wheat (Ali et al., 2015). In addition, Cr^VI^ toxicity can affect plant resistance (Singh et al., 2020; Daud et al., 2021). However, NaHS and Si treatments either alone or in combination increased plant growth and biomass in both chickpea varieties in the present study, suggesting synergistic roles in improving growth and biomass production in plants under Cr^VI^ stress (Figure 1). Previous studies have documented similar positive effects of NaHS + Si treatment under boron and drought stress in pepper plants (Kaya et al., 2020a) and wheat (Naz et al., 2021). In the present study, tolerance to Cr^VI^ stress was more effective in plants subjected to combined treatment (NaHS + Si); however, the increase in morpho-physiological parameters following treatment with NaHS and Si alone or in combination was higher in Pusa 2085 compared with that in Pusa Green 112.

The results of the present study also showed that reduction in plant growth due to Cr^VI^ stress decreased the concentration of photosynthetic pigments, which is attributed to ultrastructural alterations in chloroplasts (Gill et al., 2015; Singh et al., 2021) or overproduction of ROS under Cr^VI^ stress (Singh et al., 2020). In addition, NaHS and Si treatment has been shown to improve internodal elongation of shoots by promoting cell division (Raven, 1983; Hossain et al., 2007), thereby increasing growth under Cr^VI^ stress (Mandlik et al., 2020; Yang et al., 2021). Si is a growth regulator-like compound that enhances cell division in rice plants (Kabir et al., 2016); hence treatment of NaHS and Si can reduce the toxic effects of Cr^VI^ on chickpea plant growth and its physiological properties during stress. The results of the present study suggest that exogenous NaHS and Si enhanced plant tolerance by increasing photosynthetic pigments in chickpea plant leaves. Under Cr stress, NaHS treatment increases photosynthetic pigments in cauliflower (Ahmad et al., 2020), black bean, and mungbean (Husain et al., 2021). Furthermore, exogenous Si application can also improve photosynthetic performance in wheat (Sarkar et al., 2020), mustard (Ashfaque et al., 2017), and rice under Cr stress (Huda et al., 2017; Yang et al., 2021). In this study, NaHS and Si enhanced chlorophyll synthesis in both chickpea plants under Cr stress. Our results resonate with those of Naz et al. (2021), who studied wheat plants under Cr stress. The increase in photosynthetic pigment under Cr stress may be attributed to the increased plant tolerance and enhanced antioxidant properties of NaHS and Si.

In our study, chickpea plants treated with Cr^VI^ displayed severe oxidative stress in leaves and root tissues, as evidenced by the increased levels of H_2_O_2_ and overproduction of ROS (Figure 3). H_2_O_2_ plays an important role in cell signalling (Nazir et al., 2020) and is the primary ROS molecule that negatively affects plant cells (Gill et al., 2015; Yang et al., 2021). Exposure to Cr^VI^ stress induced oxidative damage in chickpea plants by disrupting the balance between ROS generation and clearance by antioxidative enzymes. Singh et al. (2021) reported a similar relationship between oxidative damage (MDA, EL, and H_2_O_2_ levels) and proline content in the roots and leaves, and enhanced antioxidant properties of leaves in mungbean plants. However, exogenous application of NaHS and Si, alone or in combination, reduced oxidative stress in roots and leaves of both varieties under Cr^VI^ stress in the present study. Furthermore, NaHS and Si enhanced membrane protection or stability, as observed by the low production of MDA and EL in the chickpea plant tissues. NaHS treatment improves the antioxidant system and plant tolerance by reducing oxidative stress in the roots and leaves of cauliflower plants which reduces cell membrane injury (Ma et al., 2016; Ahmad et al., 2020). In our study, NaHS and Si reduced oxidative damage (MDA, EL, and H_2_O_2_) in chickpea plants under Cr^VI^ stress thereby improving plant tolerance. Thus, our results on the benefits of NaHS or Si treatment under oxidative stress agree with those of previous studies on toxic effects of Cr in rice (Tripathi et al., 2012) and maize (Kharbech et al., 2020). However, evidence supporting the synergistic role of NaHS + Si in mitigating Cr^VI^ toxicity remains lacking. The results of the present study indicate that synergistic action of exogenous NaHS and Si reduces total ROS levels and enzymatic ROS generation.

Furthermore, the amount of proline in chickpea plants was enhanced upon exposure to Cr, as previously reported by Karthik et al. (2021), who observed upregulation of proline high proteins thereby protecting the cells from ferroptosis. Similar increase in proline content in the roots and leaves under Cr stress has also been reported in maize (Adhikari et al., 2020), chickpea (Singh et al., 2020), spinach (Hussain et al., 2021), and tomato (Martins et al., 2021) crops. Excessive proline accumulation under stress conditions helps plants to maintain a redox potential and osmotic activity to stabilize cellular structures and ROS scavenging (Forlani et al., 2019). Plants under stress accumulate high amounts of osmoprotectants, such as proline, to overcome cellular dehydration mediated by high uptake of heavy metals (Sharma et al., 2019; Mostofa et al., 2020). The findings of the present study confirm that chickpea plants have high proline accumulation in cope with Cr^VI^-induced water losses. Under NaHS, Si, and NaHS + Si treatment conditions, the slight increase in proline content suggests their partial involvement in proline metabolism. Moreover, NaHS and/or Si application improved leaf water status by enhancing RWC, cell dehydration, and cellular functions in chickpea plants under Cr^VI^ stress. A similar relationship was observed in Cr-treated rice plants (Yang et al., 2021). Si application under Cd stress increases proline while enhancing leaf water status and declines oxidative stress, suggesting that proline accumulation may enhance leaf water status and have a beneficial role in reversing Cd-induced oxidative stress in pepper (Kaya et al., 2020). Similarly, Si application increases proline levels in various crop species like tomato (Alam et al., 2021), pea (Jan et al., 2018), and Indian mustard (Pandey et al., 2016; Ashfaque et al., 2017) under several heavy metal stress. These results also illustrate the protective roles of NaHS and Si, alone or in combination, under conditions of water scarcity.

The exposure of chickpea plants to Cr declined RWC, a stress indicator (Pandian et al., 2020). A significant decline in RWC has been reported in various crop such as rice (Pandey et al., 2019; Yang et al., 2021) and oilseed rape (Gill et al., 2015) under Cr^VI^ stress, which is possibly attributed to the higher assimilation rate of Cr ions in roots compared to shoots, which protects shoots from high Cr accumulations by promoting optimum water intake in aerial parts (Ao et al., 2022). Similar relationships have been observed with heavy metal stress in various crop plants (Bali et al., 2019; Pandian et al., 2020). However, in the present study, the exogenous application of NaHS and Si, alone or in combination, improved RWC in the leaves of both chickpea plants. Similarly, Si application mitigate the toxic effects of Cr^VI^ by enhancing RWC *via* reducing metal accumulation (Alam et al., 2021; Yang et al., 2021), which improves plant growth and biomass production and increases the activities of antioxidant enzymes (Tripathi et al., 2015; Huda et al., 2017).

Plants under Cr stress may disturb production and elimination of ROS (Shahid et al., 2017), thereby increasing ROS production and membrane lipid peroxidation and disrupting the structure and function of the cell membrane system (Yu et al., 2017; Kushwaha and Singh, 2020a). In the present study, the enzymatic and non-enzymatic antioxidant activities in chickpea plant leaves decreased under Cr^VI^ stress, whereas DHA and GSSG levels increased. Similar results have been reported in some vegetable crops (Kushwaha and Singh, 2020a). However, application of NaHS and Si, alone or in combination, stimulated all antioxidant enzymes and enhanced the levels of non-enzymatic antioxidants, which declined the levels of DHA and GSSG declined in both varieties. Our results clearly showed that NaHS and Si increase the antioxidant defence mechanisms by reducing oxidative stress in chickpea plants under Cr^VI^ stress. These results indicate that activation of APX, DHAR, and MDHAR enzymes may prevent the excessive accumulation of ROS and enhance the tolerance capacity of plants, as reported by Yang et al. (2021). Moreover, enzymatic (SOD, CAT, GR, APX, GST, DHAR, and MDHAR) and non-enzymatic (AsA, GSH, and GSSG) antioxidant levels also increase following Si treatment in tomato plants under Cr^VI^ stress (Alam et al., 2021). Similarly, decrease in oxidative damage in different crops under Cr^VI^ stress have been reported following NaHS and Si treatment due to an increase in enzymatic antioxidant defence mechanisms (Ahmad et al., 2020; Alam et al., 2021). Recent studies have reported that NaHS and Si treatment enhanced rice plants defence mechanism against oxidative damage induced by copper oxide nanoparticles (Rai et al., 2021). In the present study, NaHS and Si regulated the AsA-GSH enzyme cycle by reducing the oxidative damage induced by the free radicals. The AsA-GHS associated defence mechanism demonstrated further enhanced levels of GHS after NaHS and/or Si treatment. The decline in GHS and DHA activities was positively correlated with AsA and GSSG enzyme levels, which may have improved the efficiencies of the AsA/DHA and GSH/GSSG enzyme cycles. A similar relationship was reported for Cd-treated rice plants (Mostofa et al., 2015). However, the application of Si maintains the redox pool of AsA and GSH, as confirmed by the increase in AsA/DHA and GSH/GSSG ratio. Our results are in line with those of Gangwar et al. (2011), who reported an increase in AsA-GHS enzyme ratio in pea seedlings under Cr stress with gibberellic acid application.

Cr^VI^ stress reduced PM H^+^-ATPase activity in leaves of chickpea plants. H^+^-ATPase is a key enzyme that regulates plants’ physiological and molecular processes by maintaining the cytosolic pH and membrane-associated electrochemical gradient (Gildensoph and Briskin, 1990). PM H^+^-ATPase activates secondary ion transport across membranes, thereby regulating several processes, such as cell growth, nutrient uptake, intracellular pH regulation, and stomatal opening (Falhof et al., 2016; Zhang et al., 2017; Siddiqui et al., 2021b). The application of NaHS and Si, alone or in combination, increased PM-H^+^-ATPase activity in the leaves of chickpea plants under Cr^VI^ stress. However, the increase in the activity was higher in the Pusa 2085 (Cr-resistant) variety than in Pusa Green 112 (Cr-sensitive) variety. Similar results have been reported for different plant species, including soybeans under Al stress (Shen et al., 2005; Kim et al., 2010; Chen et al., 2013). The results of this study suggest that increased PM-H^+^-ATPase activity can restore membrane potential and ion transport by preventing electrolytic leakage or ion leaching from leaf tissue. Our results are consistent with those reported by Khan et al. (2021), who reported the role of H_2_S in upregulating H^+^-ATPase activity and ion homeostasis during NaCl-toxicity in mungbean plants. NaHS and Si treatment also showed a decrease in MDA levels in both root and leaf tissues, suggesting a reduced extent of membrane lipid peroxidation due to decreased ROS production and improved antioxidant defence mechanism. Thus, these beneficial effects of NaHS and Si under Cr^VI^ stress and increased plasma membrane H^+^-ATPase activity may further enhance the repair processes in heavy-metal-stressed plants. Another reason behind the decline in PM H^+^-ATPase activity in chickpea plant leaves under Cr stress might be the decrease in the nutrient uptake levels, as similarly demonstrated by Singh et al. (2022).

The present study reports that exposure of chickpea plants to Cr-contaminated soils reduces plant growth and nutrient uptake, which contributes to the decline in essential nutrients. Due to the ionic resemblance with nutrients, excessive Cr levels may displace/interfere with nutrients absorption and translocation. In contrast, NaHS and Si treatment, alone or in combination, can promote the uptake of macronutrients during Cr stress, as confirmed by the improved mineral content in chickpea roots and leaves. Our results showed higher rate of increase in the concentration of these minerals in Pusa 2085 (tolerant variety) as compared to Pusa Green 112 (sensitive variety). One study on pepper plants under Boron stress revealed a protective role of NaHS and Si against boron-triggered membrane damage by reducing lipid peroxidation (as MDA) and ROS (as O_2_¯ and H_2_O_2_) production (Kaya et al., 2020a), which caused higher accumulation of nutrient contents. Our results showed that improved plant growth and better Cr stress tolerance in Pusa 2085 variety were linked to less ROS accumulation and oxidative damage, increased PM H^+^-ATPase activity, strong antioxidant defence mechanism, higher accumulation of proline, higher nutrient uptake, and lower accumulation of Cr than Pusa Green 112 variety.

The increase in nutrient uptake in roots and leaves of NaHS and Si treated chickpea plants can directly affect metabolism by regulating key functions, such as photosynthetic pigment, PM-H^+^-ATPase activity, lipid peroxidation, and enzyme regulation. This suggests that the application of both NaHS and Si work synergistically to enhance the accumulation and uptake of inorganic mineral nutrients under Cr stress, suggesting a high Cr uptake in roots and less translocation from root to shoot, which have also been reported (Zaheer et al., 2020; Ahmad et al., 2022). However, the individual or interactive effects of NaHS and Si reduced the accumulation of Cr by confining Cr to the aboveground parts of the plant. The application of Si in tomato plants during Cr stress showed similar results in plant tolerance (Gill and Tuteja, 2010). In the present study, exogenous application of NaHS and Si in Cr^VI^-treated plants revealed better growth and lower Cr uptake, thereby enhancing chickpea tolerance and defence mechanisms, which increased the nutrient content in roots and leaves. Overall, our results indicated that the combined exogenous treatment of NaHS and Si is more effective in reducing Cr^VI^ stress in both chickpea varieties than the single exogenous Si and NaHS treatments.

## Conclusion

The present study elucidated that Cr^VI^ stress in chickpea plants resulted in a decline in growth and biomass production, pigment content, RWC, essential minerals, and enzymatic and non-enzymatic antioxidants (AsA and GSH), and increased MDA content level and ROS production. However, the application of NaHS and Si, alone or in combination, in chickpea plants under Cr^VI^ stress is an effective strategy to improve plants defence mechanism and overall health. The combined treatment of NaHS and Si was more effective in detoxifying ROS by enhancing the abilities of enzymatic and non-enzymatic antioxidants to improve osmolyte production and prevent oxidative damage. Furthermore, the combined stress of NaHS and Si played an important role in maintaining the AsA/DHA and GSH/GSSG enzyme ratios, which also declined the oxidative damage by improving GSH levels. In addition, in those stress conditions, a positive correlation with the uptake of essential nutrients was observed in the roots and leaves of chickpea plants due to decreased accumulation of Cr^VI^. Furthermore, the findings of this study can be used to elucidate the chelation mechanism involved in NaSH and Si-induced metal tolerance of plants. Pusa 2085 showed increased morpho-physiological parameters and better Cr^VI^ stress tolerance as compared to Pusa Green 112, which was possibly linked to the reduction in ROS production and oxidative damage, strong correlation with antioxidant defence system, and higher accumulation of proline, PM H^+^-ATPase activity, AsA, GHS, GSSG, nutrients, and lower accumulation of Cr, compared with Pusa Green 112. The benefit/cost ratio of the application of chemicals must be investigated before recommending them for widespread use under natural field conditions.

## Data Availability Statement

The original contributions presented in the study are included in the article/**Supplementary Material**, further inquiries can be directed to the corresponding authors.

## Author Contributions

DeS: conceptualization, methodology, and writing—original draft preparation. AR, SS, and AB: data analysis and prepared all figures. DeS, MS, SA, DhS, CS, NS, SP, and HK: validation and critical review of the manuscript. All authors contributed to the article and approved the submitted version.

## Funding

This Research work was supported by the University Grants Commission (UGC), New Delhi, India and King Saud University, Riyadh, Saudi Arabia.

## Conflict of Interest

The authors declare that the research was conducted in the absence of any commercial or financial relationships that could be construed as a potential conflict of interest.

## Publisher’s Note

All claims expressed in this article are solely those of the authors and do not necessarily represent those of their affiliated organizations, or those of the publisher, the editors and the reviewers. Any product that may be evaluated in this article, or claim that may be made by its manufacturer, is not guaranteed or endorsed by the publisher.
