# Supplementary material for: Hydrogen Sulfide and Silicon Together Alleviate Chromium (VI) Toxicity by Modulating Morpho-Physiological and Key Antioxidant Defense Systems in Chickpea (Cicer arietinum L.) Varieties
Source: Front Plant Sci. 2022 Jul 22;13:963394. doi: 10.3389/fpls.2022.963394 (PMC9374685; doi:10.3389/fpls.2022.963394)
Supplement: Supplementary file 1 [file Table_1.DOCX]

**Table S1**. Effect of NaHS, Si, and NaHS + Si treatments on N-nitrogen, P-phosphorus, K-potassium, Ca-calcium, and Mg-magnesium contents in roots and leaves of both chickpea plants grown during Cr^VI^ stress. Data are presented as ± standard error (n = 5) and different bar letters are indicated statistically differences (p < 0.05) as tested by the least significant difference test.

| Varieties | Treatments | leaves (mg g^−1^ DW) | | | | | Roots (mg g^−1^ DW) | | | | |
| --- | --- | --- | --- | --- | --- | --- | --- | --- | --- | --- | --- |
|  |  | N | P | K | Ca | Mg | N | P | K | Ca | Mg |
| Pusa 2085 | Control | 6.59 $\pm$ 0.71^a^ | 4.84 $\pm$ 0.47^a^ | 32.96 $\pm$ 2.91^a^ | 17.48 $\pm$ 1.57^a^ | 3.91 $\pm$ 0.48^a^ | 5.19 $\pm$ 0.46^a^ | 3.77 $\pm$ 0.45^a^ | 21.73 $\pm$ 3.05^a^ | 14.46 $\pm$ 1.19^a^ | 2.74 $\pm$ 0.52^a^ |
|  | Cr | 3.70 $\pm$0.52^b^ | 3.11 $\pm$ 0.42^b^ | 18.95 $\pm$ 1.05^b^ | 8.99 $\pm$ 0.80^c^ | 1.92 $\pm$ 0.29^b^ | 2.02 $\pm$ 0.35^c^ | 1.42 $\pm$ 0.27^c^ | 10.42 $\pm$ 1.13^b^ | 5.71 $\pm$ 0.58^c^ | 0.81 $\pm$ 0.05^c^ |
|  | Cr + NaSH | 5.03 $\pm$ 0.59^ab^ | 4.25 $\pm$ 0.56^ab^ | 26.21 $\pm$ 2.27^a^ | 13.21 $\pm$ 1.03^b^ | 2.84 $\pm$ 0.44^ab^ | 3.09 $\pm$ 0.35^bc^ | 2.25 $\pm$ 0.29^bc^ | 16.48 $\pm$ 1.91^ab^ | 9.56 $\pm$ 1.02^b^ | 1.49 $\pm$ 0.17^bc^ |
|  | Cr + Si | 5.63 $\pm$ 0.54^a^ | 4.36 $\pm$ 0.54^ab^ | 27.01 $\pm$ 2.04^a^ | 14.21 $\pm$ 0.87^ab^ | 3.04 $\pm$ 0.60^ab^ | 3.17 $\pm$ 0.45^bc^ | 2.45 $\pm$ 0.37^b^ | 17.48 $\pm$ 2.16^a^ | 10.10 $\pm$ 0.69^b^ | 1.69 $\pm$ 0.15^bc^ |
|  | Cr + NaSH + Si | 6.63 $\pm$ 0.64^a^ | 4.73 $\pm$ 0.53^a^ | 31.01 $\pm$ 3.23^a^ | 14.76 $\pm$ 1.22^ab^ | 3.64 $\pm$ 0.61^a^ | 4.29 $\pm$ 0.67^b^ | 3.13 $\pm$ 0.21^ab^ | 19.68 $\pm$ 1.66^a^ | 12.16 $\pm$ 1.04^ab^ | 2.49 $\pm$ 0.52^ab^ |
| Pusa Green 112 | Control | 6.19 $\pm$ 0.44^a^ | 4.24 $\pm$ 0.41^a^ | 31.76 $\pm$ 2.21^a^ | 15.82 $\pm$ 1.52^a^ | 3.83 $\pm$ 0.52^a^ | 4.79 $\pm$ 0.53^a^ | 3.37 $\pm$ 0.55^a^ | 20.53 $\pm$ 2.74^a^ | 13.26 $\pm$ 1.26^a^ | 2.34 $\pm$ 0.34^a^ |
|  | Cr | 2.77 $\pm$ 0.33^c^ | 1.93 $\pm$ 0.28^b^ | 16.09 $\pm$ 1.92^c^ | 6.79 $\pm$ 0.71^c^ | 1.71 $\pm$ 0.23^c^ | 1.70 $\pm$ 0.22^c^ | 1.10 $\pm$ 0.13^b^ | 8.42 $\pm$ 0.51^c^ | 4.91 $\pm$ 0.53^c^ | 0.64 $\pm$ 0.11^c^ |
|  | Cr + NaSH | 3.56 $\pm$ 0.29^bc^ | 2.49 $\pm$ 0.35^b^ | 21.77 $\pm$ 1.51^bc^ | 9.08 $\pm$ 0.90^bc^ | 2.25 $\pm$ 0.36^bc^ | 2.17 $\pm$ 0.23^bc^ | 1.38 $\pm$ 0.18^b^ | 11.28 $\pm$ 1.06^bc^ | 6.96 $\pm$ 0.95^bc^ | 0.98 $\pm$ 0.12^bc^ |
|  | Cr + Si | 3.63 $\pm$ 0.69^bc^ | 2.66 $\pm$ 0.46^b^ | 22.31 $\pm$ 2.16^b^ | 10.41 $\pm$ 0.63^b^ | 2.50 $\pm$ 0.27^bc^ | 2.37 $\pm$ 0.23^bc^ | 1.47 $\pm$ 0.17^b^ | 12.48 $\pm$ 1.08^bc^ | 7.08 $\pm$ 0.94^bc^ | 1.01 $\pm$ 0.04^bc^ |
|  | Cr + NaSH + Si | 4.23 $\pm$ 0.49^b^ | 2.73 $\pm$ 0.45^b^ | 24.41 $\pm$ 2.43^b^ | 10.76 $\pm$ 1.01^b^ | 2.84 $\pm$ 0.44^ab^ | 3.09 $\pm$ 0.46^b^ | 1.89 $\pm$ 0.38^b^ | 14.82 $\pm$ 1.85^b^ | 7.95 $\pm$ 0.83^b^ | 1.35 $\pm$ 0.14^b^ |
| Variety (Pr > F) | | 0.0001 | <.0001 | 0.0082 | <.0001 | 0.1202 | 0.0090 | 0.0007 | 0.0037 | 0.0003 | 0.0019 |
| Treatment (Pr > F) | | <.0001 | 0.0020 | <.0001 | <.0001 | 0.0007 | <.0001 | <.0001 | <.0001 | <.0001 | <.0001 |
| Variety $\times$ Treatment | | 0.3537 | 0.5572 | 0.8013 | 0.6878 | 0.9255 | 0.8191 | 0.5914 | 0.7261 | 0.3695 | 0.4951 |
| LSD**_0.05_** | | 0.6812 | 0.5814 | 2.8785 | 1.3685 | 0.567 | 0.536 | 0.4177 | 2.3943 | 1.1945 | 0.3522 |
